# Aspects of cavity engineering in THz quantum cascade laser frequency combs

**DOI:** 10.1515/nanoph-2025-0145

**Published:** 2025-08-28

**Authors:** Lukas Seitner, Michael A. Schreiber, Michael Rinderle, Niklas Pichel, Michael Haider, Christian Jirauschek

**Affiliations:** TUM School of Computation, Information and Technology, 9184 Technical University of Munich (TUM), 85748 Garching, Germany

**Keywords:** quantum cascade laser, frequency comb, cavity engineering, Maxwell-density matrix

## Abstract

Terahertz quantum cascade laser (QCL) frequency combs are a disruptive technology for spectroscopy, imaging, and quantum technologies in this spectral range. For advanced development and tailoring of frequency comb properties, detailed modeling of the QCL device is necessary. Recent achievements in the field of THz QCLs have unveiled that custom modifications of the laser cavity by dispersion engineering or tapered waveguide sections significantly influence the laser’s behavior. In this article, we present a numerical model based on the Maxwell-density matrix formalism that captures such cavity effects in detail, yielding a better understanding of the QCL dynamics and opening the possibility of designing cavities for custom laser applications. We show that waveguide engineering in terms of dispersion compensation and field enhancement can stabilize an unlocked multimode state into frequency comb operation and even shape its properties, such as the bandwidth or the mode spacing of harmonic frequency combs.

## 1 Introduction

The terahertz portion of the electromagnetic spectrum has increasingly attracted many research activities, aiming to develop improved emitters and detectors that can potentially unlock a wide range of applications [1]. Using this spectral band for sensing, imaging, security, or communication requires high-power, portable, and ideally coherent sources. Somewhat more than 30 years ago, the quantum cascade laser (QCL) was demonstrated in the mid-infrared, potentially fulfilling these requirements [2]. The name refers to its active region design, which is based on a semiconductor heterostructure with alternating conduction band offset and engineered intersubband levels. Some years later, this technology was transferred to the THz range, with the first laser operating at ≈ 4.45 THz, corresponding to about 67 µm free-space wavelength [3]. Since then, many striking developments have taken place, where the realization of frequency comb operation is one outstanding achievement [4], [5], [6]. Short THz pulse operation can be obtained by active and passive mode locking techniques [7], [8]. However, due to fast gain dynamics in the picosecond range, free-running QCLs tend to produce rather continuous intensity patterns while self-modulating their frequency [9], [10]. Apart from these frequency-modulated (FM) combs, harmonic frequency combs (HFCs) characterized by a mode spacing of multiples of the free spectral range (FSR) are a current subject of research [11], [12], [13]. Recent developments and achievements concerning THz QCLs include, among others, an increased operating temperature, advanced mode locking techniques, meta-surface integration, and the use as a fast detector [8], [14], [15], [16], [17].

The numerical modeling of QCL frequency combs is typically based on Maxwell–Bloch equations, which couple the propagation of the electric field in one spatial dimension to the active region dynamics [18], [19], [20]. Many efforts were made to successfully describe the intersubband charge carrier transport and gain properties by rate equations, the Monte-Carlo method, or the nonequilibrium Green’s function formalism [21], [22]. Recent dynamical models cover a broad range of complexity, spanning from multilevel density matrix and full-wave field solvers to effective master equations and semi-analytic mean field theories [23], [24], [25], [26], [27], [28]. These spatiotemporal models are especially suited for including cavity modifications, such as defects, microwave copropagation, or saturable absorbers [29], [30], [31], [32], [33].

Previous theoretical work based on Maxwell–Bloch-type equations has primarily focused on the modeling of the QCL active region, while advanced waveguide structures beyond standard Fabry–Perot or ring cavities have rarely been considered. Notable exceptions include the implementation of air slits [29], an external cavity [27], or a distributed feedback grating [34]. In the present work, we discuss a general numerical model for QCLs with multiple sections, potentially featuring different refractive indices, changing waveguide widths, and complex-valued reflection and transmission coefficients. Thus, experimentally realized structures, such as Gires–Tournois interferometers [35], [36] or tapered cavities [37], can be treated consistently within the numerical Maxwell-density matrix framework [24]. With that, we unravel the influence of cavity engineering on frequency comb formation and the shaping of the temporal and spectral characteristics of the electric field. Our results bring relevant insights into dispersion engineering and field enhancement by waveguide modification and their influence on the active region dynamics. The developed model is consistent with recent experimental results and can be used for laser analysis and design in future work.

## 2 Theoretical model and numerical implementation

Usually, Maxwell–Bloch-type models for QCL simulation consist of three parts. Thereby, the propagation of the electric field is coupled to the microscopic charge carrier dynamics via the medium polarization. Finally, boundary conditions, which represent the properties of the cavity, complete the model. This section introduces the electric field model and its numerical implementation in a possibly defect-engineered or tapered cavity.

### 2.1 Electric field and medium polarization

In an actual QCL device, the electric field is a three-dimensional quantity. However, the active region only amplifies the out-of-plane component of the electric field, such that transverse magnetic modes are enforced. By using the slab waveguide approximation, i.e., assuming the transversal field distribution to be invariant along the waveguide, a one-dimensional description of the time-dependent electric field *E*(*x*, *t*) in its propagation direction *x* is sufficient to capture the laser dynamics, significantly reducing the numerical load [24]. Moreover, we assume that the field envelope varies slowly compared to the carrier frequency. In this slowly varying envelope approximation (SVEA), the field is decomposed into two counterpropagating components *E* ^±^(*x*, *t*), according to the ansatz

(1) E(x,t)=∑±ReE±(x,t)exp±iϕ−iωct,

where *ω*_c_ = 2*πf*_c_ denotes the center frequency. Furthermore, $\phi =\int_{0}^{x}\beta_{0}\left(x^{\prime }\right)dx^{\prime }$ is the global phase of the field envelopes, with the potentially space-dependent propagation constant $\beta_{0}\left(x\right)$ . In a homogeneous medium, where *β*_0_ is uniform over the entire considered space, this phase simplifies to the commonly used expression *ϕ* = *β*_0_ *x*. However, for more complex geometries with changing refractive indices, considering the full expression for the global phase is necessary. The spatiotemporal evolution of the one-dimensional fields in the SVEA is then described by the propagation equation [24]

(2) $$v_{\text{g}}^{-1}\partial_{t}E^{\pm }=\mp \partial_{x}E^{\pm }+f^{\pm }-\frac{a}{2}E^{\pm }-i\frac{\beta_{2}}{2}\partial_{t}^{2}E^{\pm }+S_{\text{sp}}^{\pm },$$

derived from Maxwell’s equations. Here, *v*_g_ denotes the group velocity and *β*_2_ is the background group velocity dispersion (GVD). Furthermore, the optical power loss is given by *a*, and the term $S_{\text{sp}}^{\pm }$ describes spontaneous emission noise [38]. The active region polarization *f* ^±^ contains the optical gain and is obtained from density matrix equations governing the intersubband dynamics [24]. To numerically solve the partial differential equation (2), we apply the Risken–Nummedal finite difference (RNFD) scheme [39]. With *ℓ* = *v*_g_ *a*/2, we obtain an explicit update equation of the electric field at the next time step as

(3) $$\begin{array}{ll} E_{m,n+1}^{\pm } & =E_{m\mp 1,n}^{\pm }\\ & \;+\frac{\Delta_{t}}{2}\left[f_{m,n}^{\pm }+f_{m\mp 1,n}^{\pm }-\left(\ell_{m,n}^{\pm }+\ell_{m\mp 1,n}^{\pm }\right)E_{m\mp 1,n}^{\pm }\right]\\ & \;+\frac{\Delta_{t}^{2}}{2}\left\{{\left[\partial_{t}f^{\pm }\right]}_{m,n}-\ell_{m,n}^{\pm }f_{m,n}^{\pm }+\ell_{m,n}^{\pm^{2}}E_{m,n}^{\pm }\right\}. \end{array}$$

Here, $E_{m,n}^{\pm }$ denote the discretized field envelopes at the spatial grid point *m* and time step *n*. The temporal step size Δ*t* is linked to the spatial grid size via Δ*x* = *v*_g_Δ*t*. Furthermore, $f_{m,n}^{\pm }$ and $\ell_{m,n}^{\pm }$ represent the discretized polarization and loss functions, which can be spatially varying and different for the two propagation directions. For the temporal derivative term *∂*_*t*_ *f* ^±^ in Eq. (3), a discretization is not necessary, as it is a direct result of the density matrix formalism. The equations of motion for the density matrix are a set of ordinary differential equations that can be solved at each spatial grid point in parallel, e.g., by applying a 3rd order Adams–Bashforth scheme [24]. The terms describing spontaneous emission $S_{\text{sp}}^{\pm }$ and dispersion *β*_2_ are not included in Eq. (3) and have to be treated separately [38], [40].

### 2.2 Reflection and transmission

Partial reflection and transmission processes of the electric field play a pivotal role in THz QCLs because the dimensions of (sub-)cavities might be in the range of the intracavity wavelength of ≈ 15–70 µm, and thus influence the operation. This opens up the possibility to engineer the waveguide for beneficial properties, such as dispersion compensation or frequency filtering. The Maxwell–Bloch-type equation system described above is complemented with explicit boundary conditions for the optical field that are, in our case, defined by the laser cavity. In a linear waveguide, we have a finite field reflection at the two facets, while a ring cavity is described by periodic boundary conditions.

#### 2.2.1 Changing refractive index

Focusing on field reflections at a material interface and considering normal incidence, the Fresnel formulas yield the reflection and transmission coefficients

(4) $$r_{12}=\frac{n_{1}-n_{2}}{n_{1}+n_{2}},$$

(5) $$t_{12}=1+r_{12},$$

with *n*_1_ and *n*_2_ being the effective refractive indices of the two materials. For the facet of a QCL laser cavity, typical values for the semiconductor material are *n*_1_ ≈ 3.3 and *n*_1_ ≈ 3.6 for mid-IR and THz QCLs, respectively. In case of air surrounding the waveguide (*n*_2_ = 1), this results in a power reflection of *R*_12_ = |*r*_12_|^2^ ≈ 0.3, such that the power outcoupling *T*_12_ = 1 − *R*_12_ ≈ 0.7. However, for metal–metal waveguides in the THz range, the reflection is typically significantly larger than expected from Fresnel’s formula (*R*_12_ ≈ 0.6 − 0.8). In that case, wave-optical effects depending on the exact geometry considerably influence the electromagnetic behavior [41]. Depending on the model requirements, *r*_12_ can be extracted from three-dimensional electromagnetic simulations or analytical models [21]. At this abrupt change from a guided mode profile to free-space propagation the power still must be conserved, such that the relation

(6) $$|t_{12}|=\sqrt{n_{1}/n_{2}\left(1-r_{12}^{2}\right)}.$$

holds, if no losses occur.

However, reflections and a changing refractive index can also happen inside the laser cavity and thus within the simulation domain. For the implementation of a reflection in the SVEA model, a phase correction term must be added to the reflection process. As the electric field is modeled by two counterpropagating complex amplitudes with counter-rotating spatial phasors [Eq. (1)], *E* ^+^ and *E* ^−^ possess a spatially dependent phase difference equal to $\exp\left(2i\phi \right)$ . Thus, when a reflection happens at the location *x* = *x*_r_, the field components (partly) change their propagation direction, and the accumulated phase difference needs to be compensated by

(7) $$E_{\text{refl}}^{\mp }=r^{\pm }E^{\pm }e^{\pm 2i\phi \left(x_{\text{r}}\right)}.$$

Here, *r* ^±^ represents the reflection coefficient for the respective field direction.

**Figure 1:**
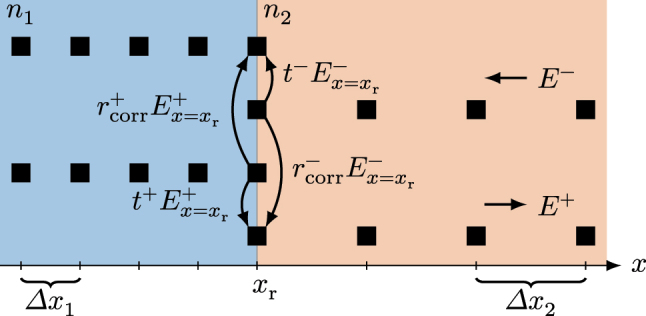
Spatial discretization of the simulation domain close to an interface of two materials with different refractive index. The two upper rows represent the grid points of the left-traveling field, while the two lower rows account for the right-traveling field. A double grid point at the interface ensures a consistent treatment of reflection and transmission processes.

Simulating several regions with varying effective refractive indices might be necessary, e.g., to model an external cavity reflector or the air gap in an integrated laser/detector setup [42]. The numerical solution of Eq. (2) using the RNFD scheme of Eq. (3) including a material interface at location *x*_r_ is illustrated in Figure 1. In this scenario, *E* ^+^ is propagating from *n*_1_ toward *n*_2_, such that the coefficient *r* ^+^ refers to *r*_12_ and consequently *r* ^−^ refers to *r*_21_. The used abbreviations $r_{\text{corr}}^{\pm }$ indicate that the phase correction factors are included in the respective reflection coefficient. Apart from the peculiarities of the reflection and transmission process, further considerations need to be taken into account. The changing refractive index in the simulation domain leads to a changing group velocity $v_{\text{g}}\left(x\right)=c_{0}/n\left(x\right)$ , with the vacuum speed of light *c*_0_. Thus, a different numerical discretization is necessary. For the implementation, it is necessary to keep the temporal grid size Δ*t* constant and vary the spatial grid size Δ*x* = *v*_g_Δ*t*, as visualized in Figure 1. Furthermore, to apply the boundary conditions from Eq. (7), the position of the interface must be chosen to coincide with a double grid point, coupling the spatial grids in both materials. By doing so, the field envelopes of the different regions are connected via the above-discussed interface conditions. Thus, the RNFD update equation (3) can be solved in each material segment individually. Since the implementation of the matching conditions, given in Eq. (7), is straightforwardly possible without extrapolating the field quantities, the numerical stability remains unaffected.

**Figure 2:**
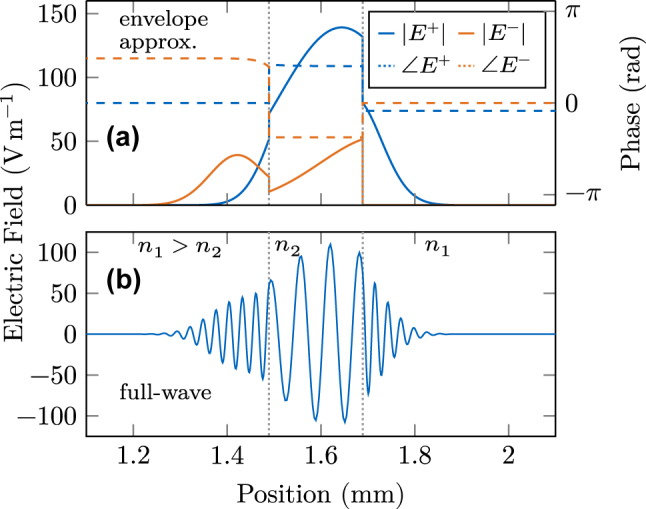
Spatially resolved electric field at a double material interface. (a) Amplitude and phase of both field components in the envelope approximation showing nontrivial behavior. (b) The reconstructed full-wave electric field at the interface is continuous but changes amplitude and frequency according to the refractive index change.

The interface implementation is tested by a double interface in a ring cavity, where we can easily compare reflected and transmitted portions of the initial pulse after several round trips. The envelope approximation introduces the same type of phase error when modeling ring cavities. At the beginning and end of the simulation domain, i.e., where the ring gets closed, the full-wave electric field needs to be continuous, such that the complex envelopes at that point need to be phase-corrected by

(8) $$E_{x=0}^{+}=E_{x=L}^{+}e^{\text{i}\phi \left(L\right)},$$

(9) $$E_{x=L}^{-}=E_{x=0}^{-}e^{\text{i}\phi \left(L\right)},$$

where *L* is the circumference of the ring. As a numerical example, we use a 200 µm section of the polymer benzocyclobutene (BCB) with refractive index *n*_2_ ≈ 1.57, used for planarized THz QCL platforms [43], in between two GaAs regions with *n*_1_ = 3.6. In Figure 2, the pulse is visualized while partially reflected and transmitted at the material interfaces. In (a), the amplitudes and phases of the right and left traveling components are shown, as calculated in the SVEA, and behave rather unintuitively due to the phase correction terms. However, from Figure 2b, it becomes clear that the correction terms are essential to be considered at interfaces in the SVEA. There, the full-wave electric field reconstructed by Eq. (1) is plotted, showing the expected behavior with phase continuity, longer wavelength, and increased amplitude due to the reduced refractive index.

**Figure 3:**
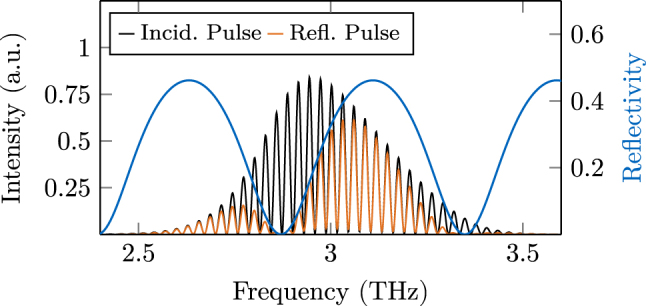
Reflected and transmitted pulse spectrum after interacting with a double facet, compared to the analytically calculated reflectivity. The correct frequency dependence is obtained by applying the phase correction terms outlined in the main text.

To validate the numerical implementation, we analytically calculate the frequency-dependent reflection and transmission coefficients for electromagnetic propagation through a thin gap by [44]

(10) $$r_{\text{gap}}=\frac{r_{12}+r_{21}\exp\left(2i\theta \right)}{1+r_{12}r_{21}\exp\left(2i\theta \right)},$$

(11) $$t_{\text{gap}}=\frac{t_{12}t_{21}\exp\left(i\theta \right)}{1+r_{12}r_{21}\exp\left(2i\theta \right)},$$

where $\theta =\left(2,\pi ,/,\lambda \right)L_{\text{gap}}$ is the phase shift due to the gap with length *L*_gap_ and the material wavelength *λ*. In Figure 3, the analytically calculated reflectivity is plotted along with the spectra of the transmitted and reflected portions of the pulse after two round trips in the defective ring cavity. We observe that the numerical implementation of the interfaces behaves correctly, as the spectrum of the reflected pulse follows the analytically calculated, frequency-dependent reflectivity very well. Without incorporating the phase correction terms of Eqs. (7)–(9), a spectral power distribution inconsistent with the analytical formula is obtained. This emphasizes the importance of the phase correction terms, especially when designing waveguides by numerical modeling. Another example for impactful reflection processes are FM combs, where the phase dynamics at the cavity facets are crucial for self-locking of the multimode field [26]. Therefore, even in scenarios where the distance between two reflections is about three orders of magnitude larger than the wavelength, a detailed modeling of interfaces is important.

#### 2.2.2 Changing waveguide geometry

One advantage of the SVEA model is the possibility to include effective reflection and transmission coefficients beyond the Fresnel relations, which is not straightforwardly possible in full-wave simulations. Therefore, one can include reflections in the one-dimensional model, which are not related to a refractive index change along the propagation direction. For example, the waveguide width might abruptly increase to realize contacting pads in a ring cavity [45]. In that case, the effective refractive index will only vary minimally, while strong field reflections occur at the sudden impedance change. Moreover, those pads present facets to the air, where significant outcoupling occurs that might be relevant for the model. For such a scenario, *r* needs to be determined individually, and the transmission coefficient can be calculated by *t* ^±^ = 1 + *r* ^±^ − *l*, where $l\in \left[0,1\right]$ represents a possible loss through outcoupling. However, when using this formula, the reflection coefficients should be chosen antisymmetrically such that *r* ^±^ = −*r* ^∓^, to avoid unphysical field amplification through a transmission larger than one. Another engineering possibility that can be included in the SVEA model is slit- or gap-type sections with a different refractive index and a length significantly shorter than the wavelength, such that they can be assumed to be frequency-independent. The corresponding reflection and transmission coefficients can then be obtained by Eqs. (10) and (11), which are generally complex, such that a phase jump at the interface correctly reproduces the phase shift of the short section. The scenario of a small gap has been successfully used to engineer Gires–Tournois interferometers for dispersion compensation or to enforce HFC operation by introducing slits in the waveguide [29], [31], [35], [36], [46]. Moreover, reflection coefficients might in general also obtain an intensity dependence, which can be experimentally realized in the THz, e.g., by using graphene [47], [48], but a detailed model of such effects exceeds the scope of this paper.

### 2.3 Tapered waveguide sections

**Figure 4:**
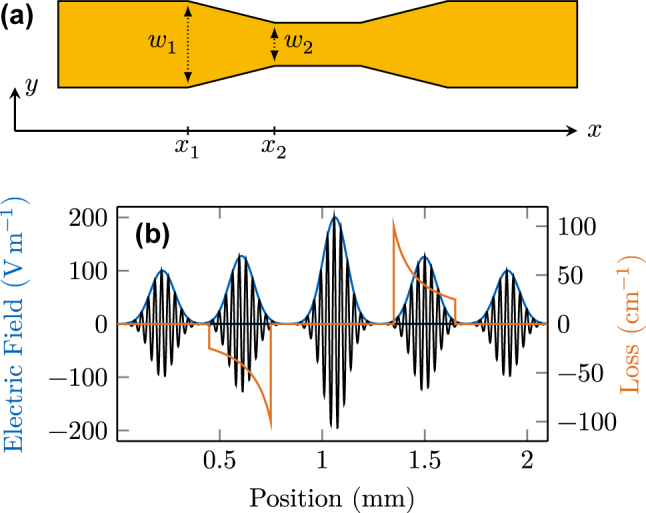
Tapered waveguide sections for field enhancement. (a) Top view of a tapered laser cavity. (b) Gaussian pulse propagating through the tapered cavity, seeing consecutive intensity gain and loss.

Apart from abrupt changes in the refractive index or the cavity geometry, the waveguide can be designed to change its width gradually, leading to field enhancement by the tapering effect. Thereby, the transverse mode profile can be assumed to adapt adiabatically, such that it is directly compatible with the one-dimensional model. We include the taper by an effective position-dependent intensity loss coefficient *a*_t_(*x*), which can also act as an amplification for *a*_t_ < 0. In the propagation equation (2), it is considered by adding *a*_t_ to the overall optical power loss *a*. For an adiabatic linear taper, we obtain (see Appendix)

(12) $$a_{\text{t}}^{\pm }\left(x\right)=\pm \frac{\left(\alpha -1\right)}{\left(x_{2}-x_{1}\right)+\left(\alpha -1\right)\left(x-x_{1}\right)},$$

where *α* = *w*_2_/*w*_1_ is the tapering factor, i.e., the ratio between the waveguide width at the end (*w*_2_) and the beginning of the tapered section (*w*_1_). Furthermore, *x*_1_ and *x*_2_ refer to the position in the field propagation direction where the tapering starts and ends, respectively. The layout of a tapered cavity device is visualized from the top view in Figure 4a, along with the relevant parameters.

To test the implementation, we seed a 1 ps pulse in a cavity without gain medium and observe its propagation behavior. We choose a tapering section with *α* = 0.25 for field enhancement and another one with *α* = 4 for consecutive amplitude reduction, both with a length of 300 µm. As $E^{\pm }\propto \sqrt{I^{\pm }}$ , the four-fold intensity increase leads to a doubling of the electric field strength, as can be observed in Figure 4b, where we plot several time frames of the pulse propagating through the tapered cavity. Furthermore, the nonlinear effective loss coefficient realized by the taper shows a maximum amplitude of ≈ ∓ 100 cm^−1^.

In conclusion, the linear tapering of a waveguide can be included in numerical simulations rather easily but can have a significant influence on QCL operation and its coherence, as we will discuss in Section 3.3.

## 3 QCL dynamical simulations

In this section, we apply the previously discussed cavity engineering techniques to QCL simulations and observe their influence. We show that careful design can lead to phase locking of an otherwise unlocked multimode state. As a first step, we develop an active region description suitable for our model.

### 3.1 Active region design

**Figure 5:**
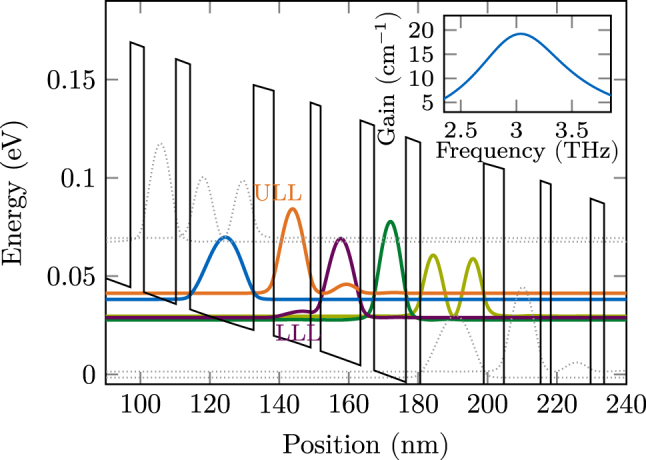
Conduction band profile and probability densities of the used QCL active region at an applied bias of 6 kV cm^−1^. The inset shows the power gain obtained from EMC simulations at this bias.

A QCL active region consisting of a multi-quantum-well heterostructure achieves optical gain by field interaction with intersubband transitions in the conduction band. Population inversion between the upper and lower laser levels (ULL/LLL) is ensured by carefully designing the heterostructure’s layer sequence and applying a suitable bias. We include the effect of this gain medium into our model by coupling the corresponding density matrix equations in the rotating-wave-approximation (RWA) to the propagation equation (2), via the polarization term *f* ^±^ [24], [49]. Required parameters include energy levels, coupling energies of tunneling transitions, dipole moments of relevant optical transitions, scattering rates between all considered levels, and dephasing rates, which are directly related to the gain bandwidth. As active region design for our simulations, we choose a four-well THz structure with a highly diagonal lasing transition, which has recently been used in tapered waveguide experiments [37], [50]. We identify five relevant energy levels per period by solving the Schrödinger–Poisson equation. The resulting probability densities are visualized in Figure 5, where the wavefunctions of energetically close states are transformed to a localized basis [51]. Furthermore, we use an ensemble Monte Carlo (EMC) simulation approach with density matrix corrections to solve the Boltzmann transport equation for the charge carrier distribution [21], [52]. The obtained spectral gain is shown in the inset of Figure 5, for a bias of 6 kV cm^−1^ at a lattice temperature of 80 K. We find that the gain dynamics features a recovery time of *τ*_g_ ≈ 3 ps, and pronounced tunnel coupling among closely aligned states is present. Therefore, the five-level system with anticrossing energies in the Hamiltonian cannot be unambiguously reduced to two levels, and we solve the full density matrix equations.

**Figure 6:**
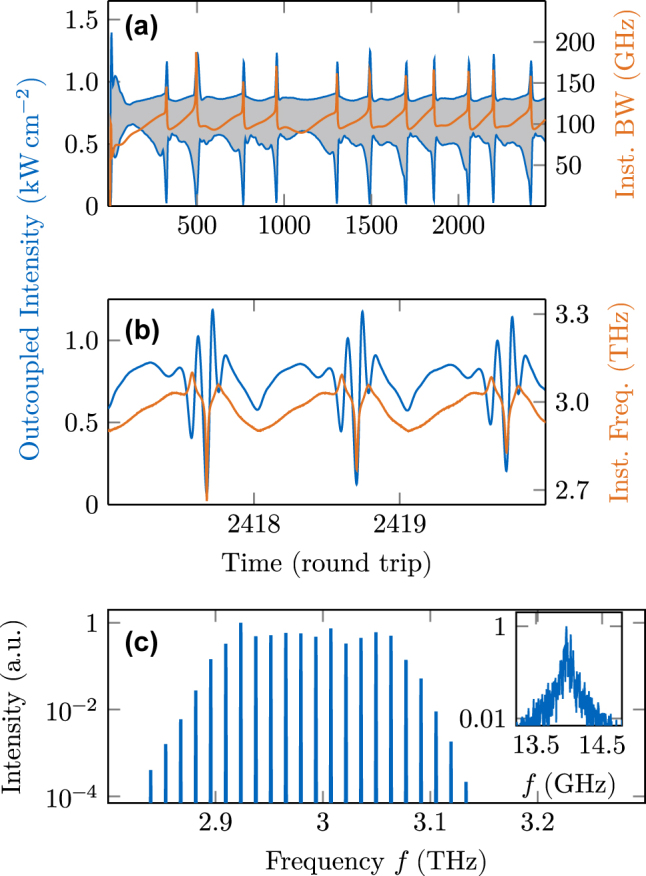
Dynamical simulation results of the active region description embedded in a pristine cavity, showing an unlocked multimode state. (a) Intensity maxima and minima of each round trip (blue) and instantaneous bandwidth (orange). (b) Intensity and instantaneous frequency over three round trips. (c) Spectrum and beatnote.

When we apply such an advanced active region model to a pristine cavity of length 2.9 mm, we obtain an unlocked state, as visible in Figure 6. The time trace of the outcoupled intensity is visualized in (a), where the upper and lower blue lines refer to the intensity maxima and minima of each round trip, respectively. The gray shaded area consequently indicates the intensity modulations. Considering a typical cross-sectional area of 1,000 µm^2^ for THz QCLs, the outcoupled power here is ≈ 7 mW. The orange line represents the instantaneous bandwidth, which we define as the maxima and minima difference of the instantaneous frequency in each round trip. For better visibility, we plot the root-mean-square (rms) value of the instantaneous bandwidth in this work, as it reduces potentially large spiking. Both signal components strongly vary over many round trips in an irregular pattern, indicating an unlocked multimode laser state. In Figure 6b, three round trips are shown at the end of the simulation time. During this short time frame, spiking and nonrepetitive intensity and frequency modulations are present. The spectrum of the last half of the simulation time is shown in Figure 6c, along with its fundamental beatnote in the inset. A rather broad spectrum with several almost equally strong modes results from Fourier transforming the electric field in the sampled time frame. The beatnote, which we obtain as the Fourier transform of the intensity, can be used as a spectral indicator for phase coherence. It exhibits a linewidth of several hundred MHz, confirming an unlocked laser state. To obtain a quantitative assessment of the multimode coherence, we apply the amplitude and phase noise quantifiers, *σ*_P_ and *σ*_ΔΦ_, respectively, to the simulated field [53]. Both values *σ*_P_ = 0.783 mW and *σ*_ΔΦ_ = 0.755 rad exceed the threshold values *σ*_P_ = 1 × 10^−2^ mW and *σ*_ΔΦ_ = 2 × 10^−2^ rad given in [53] by far, confirming the unlocked nature of the state visible in the time trace and beatnote of Figure 6.

### 3.2 Phase locking by dispersion engineering

In the following, we apply a GTI for compensation of the excessive internal GVD imposed by the gain medium [24], to achieve stable operation. Furthermore, this type of resonator leads to a filtering effect at one end of the cavity as a frequency-dependent facet reflectivity is introduced.

**Figure 7:**
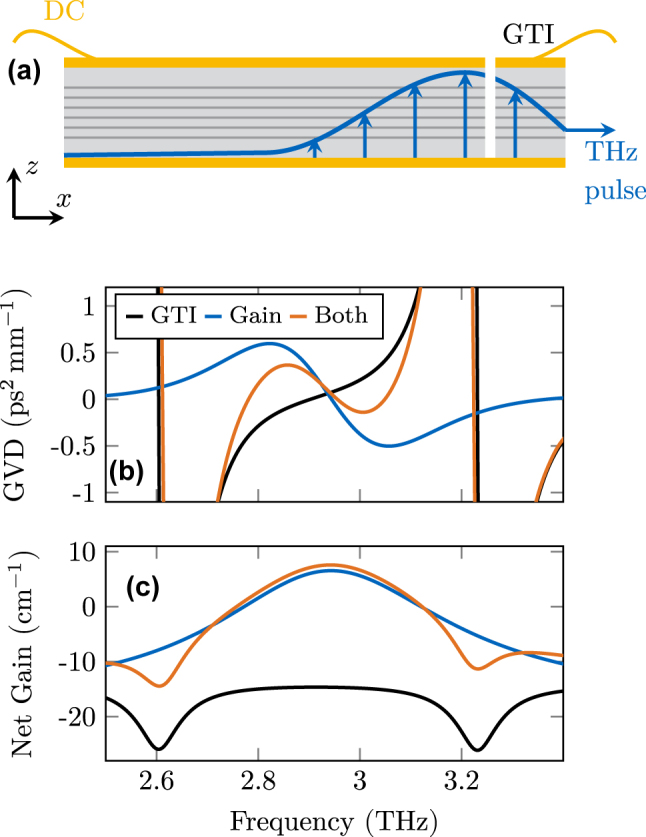
Dispersion engineering using a Gires–Tournois interferometer. (a) Illustration of a THz QCL with a GTI, probed by a THz pulse for its characterization. (b) Group velocity dispersions of a GTI with 65 µm length and 2 µm gap, of the gain medium, and their sum. (c) Net gain properties of the investigated device with and without GTI compared to the overall loss (waveguide + facets) in the presence of the GTI.

Such a GTI can be realized by etching an air gap in the active region, as has been done in an experimental study of actively mode-locked THz QCLs [36]. We assume a gap with length *L*_gap_ = 2 µm between a large and a short waveguide part, representing the GTI. This type of setup is schematically shown in Figure 7a. As the air gap is significantly shorter than the wavelength of the center frequency in free space, we model it as a frequency-independent interface with complex reflection and transmission coefficients to account for the phase shift. The reflection coefficient from the waveguide to the air *r*_12_ = 0.83 has been extracted from a numerical electromagnetic simulation [36], and from the air to the waveguide *r*_21_ = −0.83. Then, we obtain from Eqs. (10) and (11) the complex reflection and transmission coefficients of the air gap as *r*_gap_ = 0.3089 − 0.4563i and *t*_gap_ = 0.6910 + 0.4678i at a center frequency of 2.96 THz. To better understand the behavior of this rather complex GTI structure, we numerically investigate its influence on a broadband Gaussian pulse by comparing it before and after interaction with the GTI. From this pump–probe type simulation, the net gain and dispersion of the system can be retrieved by comparing the spectra of the incident and reflected pulse [49]. By including the GTI into the numerical RNFD scheme as a spatially extended structure with partially reflecting and transmitting interfaces, the frequency dependence of the GVD in the relevant bandwidth is fully captured in our model. In addition to the simulation with both the GTI and the active region, we also simulate once with only the GTI and once with only the QCL gain medium. The numerical results for the GVD and spectral net gain of a setup with GTI-length *L*_GTI_ = 65 µm and a waveguide loss of 15 cm^−1^ are shown in Figure 7b and c, respectively. We observe that the GTI significantly reduces the GVD introduced by the five-level QCL gain medium in the relevant frequency range of ≈ 2.8 THz to 3.1 THz. Apart from compensating GVD, a GTI also introduces higher-order dispersion, which might affect the laser operation. These contributions are intrinsically included in the full numerical treatment of the GTI structure, and thus, their effects cannot be investigated individually. On the other hand, our general modeling approach allows the implementation of even more complex structures for further refined dispersion engineering. In all simulated setups, an additional second-order background material GVD *β*_2_ = 0.03 ps^2^ mm^−1^ is included unless otherwise stated.

Equally important for the laser operation is the influence of the GTI on the overall net gain. While the air gap is in first approximation frequency-independent, the length of the short active region section is in the range of 2–3 wavelengths and thus introduces a strong frequency dependence. This is very well visible in Figure 7c. In the presence of the GTI, but without a gain medium (black line), the system shows largely increased loss at ≈ 2.6 THz and 3.2 THz. At these frequencies, the system consisting of the air gap and the short GaAs section is highly transparent, leading to significantly enhanced outcoupling loss. On the other hand, at the center frequency ≈ 2.96 THz, the reflection is maximized, and thus, the loss is even smaller than for the pristine cavity (compare blue and orange lines). Such loss shaping can beneficially influence THz QCL comb operation, as has also been demonstrated with a different type of cavity engineering [54].

**Figure 8:**
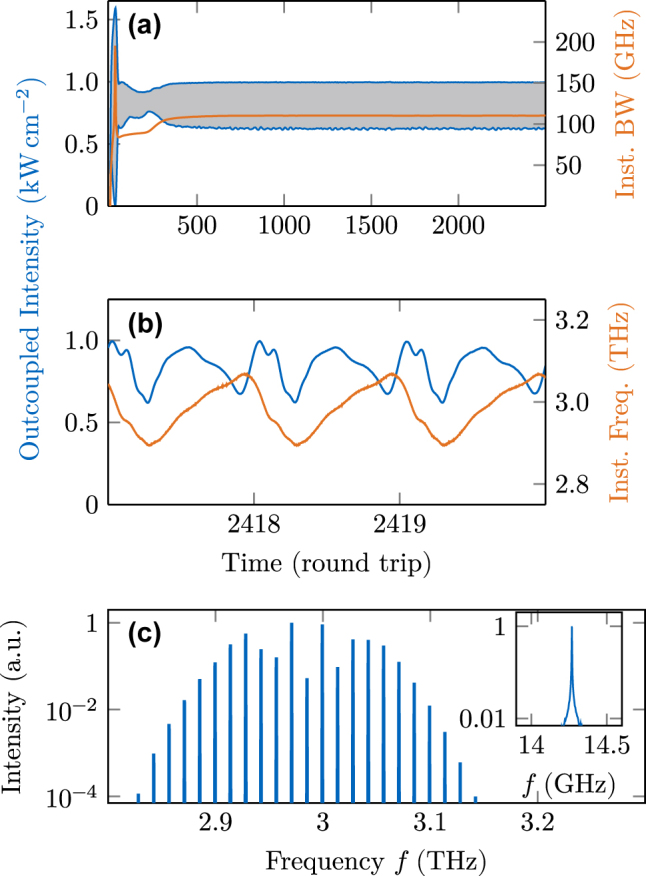
Dynamical simulation results in a cavity engineered with a Gires–Tournois interferometer. (a) Intensity and bandwidth over 2500 round trips. (b) Intensity and instantaneous frequency over three round trips. (c) Optical spectrum and beatnote.

In the here described case, the dispersion reduction and filtering effects yield phase-locked multimode operation, as confirmed by a long-term simulation including the GTI and an unchanged active region model. The results are shown in Figure 8. In (a), we observe that the intensity maxima and minima are stable over many round trips and the bandwidth remains constant with time. The transient behavior occurring in the first ≈ 300 round trips is similar to the pristine cavity case (Figure 6a), showing a significant overshoot of intensity and bandwidth. However, in the presence of a GTI (Figure 8a), no intensity bursts occur subsequently, but instead, a repeatable and coherent pattern is achieved within ≈ 100 additional round trips. A small ripple in the intensity is still visible especially for the lower intensity boundary due to the high spontaneous emission noise in THz QCLs, presenting a fundamental limitation of frequency comb coherence. In Figure 8b, the time evolution of the outcoupled signal is plotted over three round trips, showing hybrid amplitude and frequency modulation typical for THz QCL combs. While the intensity time trace appears to be somewhat arbitrary, the inherently coupled instantaneous frequency shows a largely linear chirp. Thereby, the up-chirp is longer, covering ≈ 75 % of the round trip time, compared to the down-chirp covering ≈ 25 %. The spectrum obtained from this simulation is plotted in Figure 8c. It shows two lobes, which are symmetric around the center frequency, as also observed experimentally for this active region design [50]. The simulated beatnote linewidth is limited by the numerical resolution of the Fourier transform even after 25 000 round trips and is significantly below 1 MHz. The coherence indicators *σ*_P_ = 0.0070 mW and *σ*_ΔΦ_ = 0.0058 rad confirm the presence of phase locking, despite the intense spontaneous emission noise of THz QCLs [38], [55].

### 3.3 Phase locking by field enhancement

In the previous subsection, we discussed the possibility that cavity engineering regarding GVD and frequency-dependent loss can stabilize an unlocked multimode operation of a THz QCL into an optical frequency comb. Thereby, dispersion engineering affects both the amplitude and phase of the field, according to Eq. (2). In contrast, a tapered waveguide acts on the intensity only. Therefore, the question arises whether this is sufficient to lock the modes and, if so, how the taper interacts with the gain medium to stabilize comb operation.

#### 3.3.1 Single tapered cavity

**Figure 9:**
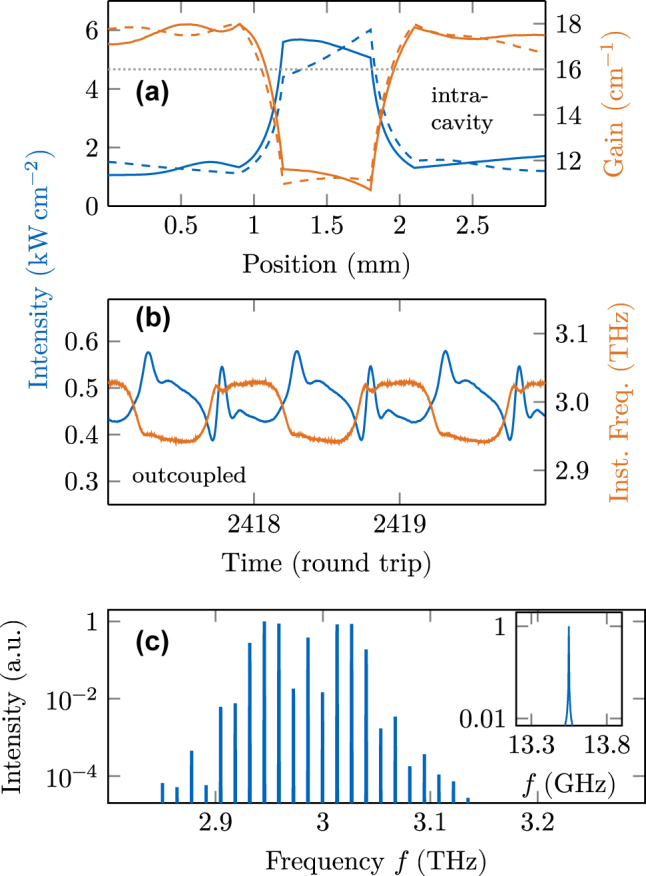
Dynamical simulation results in a cavity engineered by a tapered section. (a) Intracavity intensity (blue) and gain (orange). Solid lines refer to right-traveling properties, and dashed lines to left-traveling ones. (b) Outcoupled intensity and instantaneous frequency over three round trips. (c) Optical spectrum and beatnote.

The first question can be straightforwardly tackled by our numerical simulation approach, where we take a cavity with the layout as shown in Figure 4a. We choose the length of both tapers to be 300 µm and the narrow section in between 600 µm, while the two broad outer sections are both 900 µm, such that the overall cavity length adds up to 3 mm. The tapering factors are obtained from Eq. (12) as *α*_1_ = 0.25 and *α*_2_ = 4, respectively. As discussed in Section 3.1, an untapered device with the given active region model and waveguide length results in an unlocked state. When performing the simulation including the described taper, an optical frequency comb is obtained, characterized by the noise quantifiers *σ*_P_ = 0.0080 mW and *σ*_ΔΦ_ = 0.0065 rad, and a beatnote linewidth below our numerical resolution limit < 1 MHz. Therefore, engineering a tapered waveguide section, which leads to field enhancement, can stabilize frequency comb operation in QCLs. However, the question about the underlying dynamics remains, as a higher intensity should interact more strongly with the present nonlinearity [37] and, therefore, even enhance intensity fluctuations of an unlocked state, such as shown in Figure 6. To further investigate the taper-induced phase locking, we plot the intracavity intensity together with gain and loss in Figure 9a. It is visible that outside the tapered sections, there are regions of net gain, but in the narrow section of the waveguide, significant net loss is present. However, when calculating the average gain inside the whole cavity, we obtain $g_{\text{avg}}^{\pm }=1/L_{\text{cav}}\int_{0}^{L_{\text{cav}}}g^{\pm }dx\approx$ 16 cm^−1^, which is equal to the waveguide loss including the facets. Consequently, the laser is in a steady state, even though the gain is not saturated equally throughout the whole cavity. Due to the presence of the net loss region inside the tapered section, the formation of excessive intensity peaks, such as the ones in the bursts of Figure 6a, gets significantly more damped than in a pristine cavity. Additionally, the intensity dips associated with a burst get amplified by the provided net gain regions outside the tapered sections. Therefore, the tapered cavity design leads to a negative feedback from the amplification system on the intensity evolution, which suppresses the formation of strong fluctuations and thus supports frequency comb formation.

In the time trace of this simulation, plotted in Figure 9b, two dominant observations can be made. First, the intensity modulation amplitude is with ≈ 25 % significantly smaller than the one with GTI, where it is almost 50 %. Second, the instantaneous frequency switches extremely rapidly between two slightly chirped components, where the spontaneous emission noise is visible. We assume the switching is due to the rapid intensity enhancement in the tapered sections, and the amplitude-phase coupling of the intersubband transitions maps it to the instantaneous frequency. The rapid change between two frequency states is also reflected in the spectrum, plotted in Figure 9c. Two lobes are still visible, with significantly reduced bandwidth and one mode with medium intensity in the center. The beatnote linewidth stays below the numerical resolution with regard to its full-width-half-maximum value. Further simulations indicate that less strong tapering, i.e., with *α* closer to unity, can lead to a broader spectrum. However, a trade-off must be made with respect to the stabilizing effect, such that the field enhancement stays strong enough to keep the modes locked.

#### 3.3.2 Double tapered cavity

**Figure 10:**
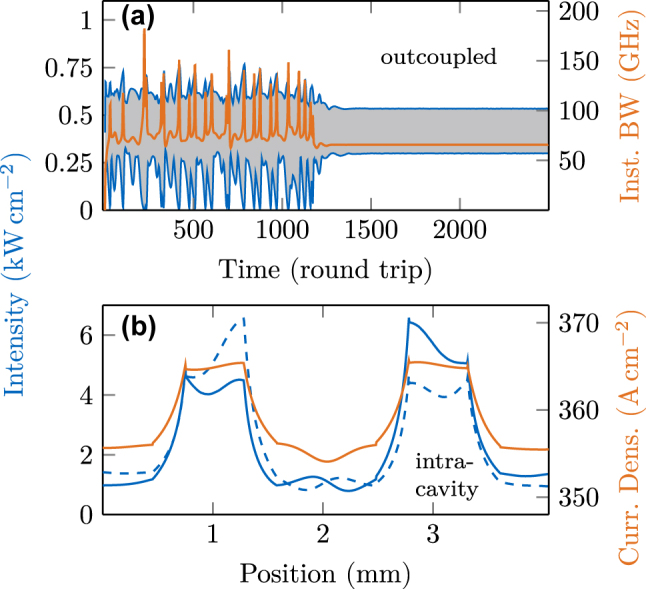
Dynamical simulation results in a cavity engineered by two tapered sections for a 2nd order HFC. (a) Outcoupled intensity maxima and minima (blue) and bandwidth (orange) over many round trips. (b) Intracavity intensity (blue) and current density (orange). The solid blue line refers to the right-traveling field, and the dashed line to the left-traveling one.

Finally, we apply our model to an experimentally realized device [37]. To this end, we extend the previously used tapered device with another taper section of the same dimensions. The section length of the first and last broad regions is reduced to 450 µm, such that the overall length of the device is ≈ 4.2 mm, as in the experiment. Furthermore, we add a small reflection coefficient *r* = 0.02 at every section interface where the waveguide dimensions change, as described in Section 2.2.2. Additionally, we increase the background GVD of the two narrow sections between the taper to *β*_2_ = 0.1 ps^2^ mm^−1^, to account for the changing propagation properties in more detail. In Figure 10, simulation results of this setup are visualized. Again, we analyze the data by monitoring the intensity and bandwidth over many round trips, as shown in Figure 10a. An unlocked and seemingly chaotic behavior dominates the dynamics for roughly 1200 round trips until a transition to a locked state happens rather abruptly. This behavior is in contrast to the transient evolution of the laser when locked by the GTI (see Figure 8a), where the output field takes its final state rapidly after reaching its saturated intensity. In Figure 10b, the left and right traveling intensity components are depicted with two clear enhancements at the two positions of waveguide narrowing. In addition, the spatially resolved current density is visualized, revealing that the tapering leads to an increased photon-driven contribution.

**Figure 11:**
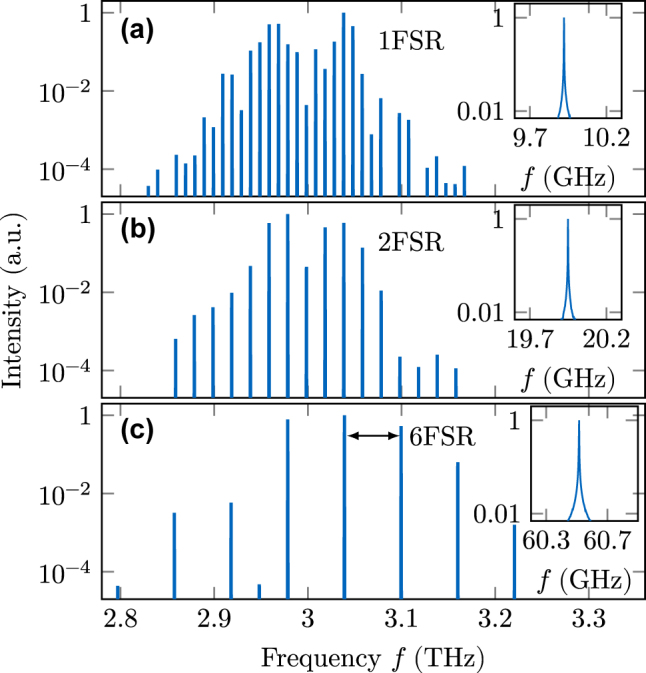
Exemplary output spectra of the double tapered cavity for different biases and temperatures. Harmonic frequency combs of different order can arise for the same cavity, with (a) and (b) even resulting from identical simulation parameters.

In experiments with such a double tapered QCL device, multiple HFCs with varying mode spacing were found, but so far not numerically modeled, to the best of our knowledge [37]. Indeed, the simulation shown in Figure 10 results in a 2FSR HFC. Due to the symmetry of the double tapered cavity and the introduced reflections, this behavior is not surprising, as symmetrically engineered cavities support HFC operation [31]. For further investigation, we use the EMC approach to extract several active region descriptions at different biases and temperatures from the QCL design given in Figure 5. We obtain mode spacings that range from a dense comb to the 6th harmonic state with the same cavity properties, as also observed in experiment [37]. Exemplary spectra are depicted in Figure 11a–c, with the lowest present beatnote of the modes given in the according inset. The 6FSR state reaches the maximum bandwidth with modes in a range of ≈ 500 GHz above the experimental noise floor (intensity range of ≈ 40 dB), as visible in Figure 11c. Interestingly, only two bias points of the QCL active region are necessary to obtain these three different comb states within the same cavity. Notably, the states of Figure 11a and b result from the same simulation setup, and the steady-state solution is selected by the randomness of the spontaneous emission noise during laser buildup. Such bistable behavior has for example been experimentally observed in mid-infrared QCLs, where dense and harmonic comb states occurred at the same bias in dependence of the current sweep direction [56]. As the results in Figure 11a and b have approximately equal output power, it is suitable to compare their coherence quantifiers. Regarding the power noise, we obtain *σ*_P,1FSR_ = 0.0052 and *σ*_P,2FSR_ = 0.0025, while the phase noise quantifiers are *σ*_ΔΦ,1FSR_ = 0.0062 and *σ*_ΔΦ,2FSR_ = 0.0030. Since the average modal power is higher for harmonic operation, the four-wave mixing is increased and thus strengthens the locking between the modes.

**Figure 12:**
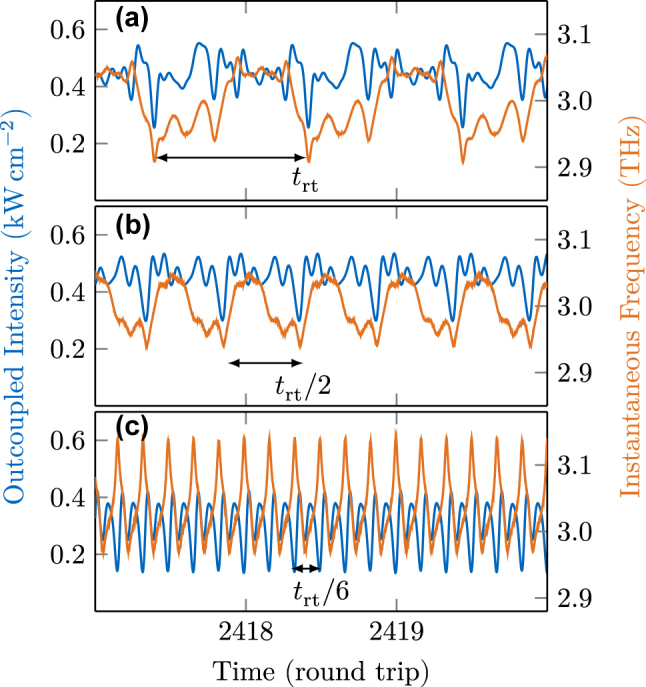
Time traces and instantaneous frequencies of the different comb states shown in Figure 11.

The time traces of intensity and instantaneous frequency associated with the spectra of Figure 11 are plotted in Figure 12a–c over three round trip times *t*_rt_. In all cases, hybrid amplitude and frequency modulation is observed, typical for THz QCLs [57], [58]. The periodicity is reduced according to the inverse of the mode spacing. In the fundamental case of Figure 12a, the instantaneous frequency chirps from its minimum to the maximum value with significant wiggles and then rapidly back to the minimum, in agreement with the experimental observations [37]. The amplitude fluctuations become more regular with increasing repetition frequency of the modulation in higher harmonic combs. Thereby, the instantaneous frequency in Figure 12c chirps nearly linearly between the two extremas, with approximately equal up- and down-chirp times.

The outcoupled power is with 3–5 mW slightly higher than found experimentally when assuming an active region cross-section of 1,000 µm^2^, which might be explained by the THz detection efficiency and the large opening angle of the radiation. The current density varies from 110 A cm^−2^ to 360 A cm^−2^. These values are a little lower than the measured range (≈ 200 A cm^−2^ to 400 A cm^−2^) [37], so the electric current is more efficiently converted to an optical field in the simulation than in the real device. Since parasitic effects, such as leakage current, are neglected in the model, this slight underestimation is well explainable.

The fact that multiple harmonic comb operations are highly stable in a cavity with a single symmetry in propagation direction is somewhat unexpected, given the fact that custom engineered HFCs of previous work closely followed the cavity structure [29], [31]. A reasoning might include the dynamic interplay between the active region properties, such as the gain bandwidth and recovery time, or tunneling energies, with the cavity dimensions. A more detailed analysis exceeds the scope of this paper, but will be part of our future work.

## 4 Summary and conclusion

In summary, we have presented a dynamical model for QCLs that accounts for reflection and transmission processes, including propagation in several media of varying refractive indices. The numerical implementation, considering phase correction factors and varying grid spacing, is validated against the analytical reflectance formula for a double-material interface. Furthermore, we have derived a model to include tapered waveguide sections in the form of an intensity gain or loss, depending on the shape of the taper and the field propagation direction. To investigate the impact of cavity engineering on THz QCL operation, we obtain an active region description by self-consistently solving the Schrödinger–Poisson and Monte-Carlo rate equations for an experimentally realized semiconductor heterostructure. Simulating this structure in a pristine cavity yields an unlocked multimode state and forms the starting point of our cavity engineering approach. At first, we show that including a GTI, which can be monolithically integrated into the waveguide, compensates for excessive GVD of the active region and thus enables frequency comb formation. Then, it is discussed that introducing a single tapered section to an otherwise pristine ridge waveguide can also have a beneficial influence on phase locking due to its peculiar interplay with the gain medium. Our simulations show that HFC formation in double-tapered waveguides is not necessarily linked to the symmetries of the cavity. We have reproduced the harmonic state switching observed experimentally, making these exotic frequency comb types more accessible. An advantage of our full numerical multilevel model is that it can be used for a wide range of QCL active region designs, including mid-infrared devices [30], [59]. Our future work will aim to better understand the occurrence of HFCs and investigate the influence of GTIs and tapered sections on actively mode-locked QCLs. Finally, we conclude that engineering the cavity of a THz QCL can enable multimode phase locking and frequency comb formation and even be used to shape the coherent field properties.

## Appendix

### Derivation of tapering loss function

We consider a tapered region between *x*_1_ and *x*_2_. Assuming an adiabatically changing transverse mode profile along the taper, we show that in the 1D model the associated intensity change can be equivalently modeled by an effective loss coefficient *a*_t_. We describe the width ratio along the tapered section between *x*_1_ and *x*_2_ by an arbitrary function

(A.1)
$$f\left(x\right)=w\left(x\right)/w\left(x_{1}\right).$$

Multiplying Eq. (2) with ${\left(E^{\pm }\right)}^{*}$ and taking the real part, we obtain the equation for the evolution of the intensity *I* ^±^∝|*E* ^±^|^2^ as

(A.2)
$$\partial_{x}I^{\pm }\pm v_{\text{g}}^{-1}\partial_{t}I^{\pm }=\cdots \mp a_{\text{t}}^{\pm }I^{\pm }.$$

Introducing the retarded time variables *t*_R_ = *t* ∓ *x*/*v*_g_, *x*_R_ = *x* yields

(A.3)
$$\partial_{x}I^{\pm }=\cdots \mp a_{\text{t}}^{\pm }I^{\pm }.$$

On the other hand, from Eq. (A.1) we obtain the taper-induced change in intensity $I^{\pm }\left(x\right)=I^{\pm }\left(x_{1}\right)/f\left(x\right)$ , and differentiation yields $\partial_{x}I^{\pm }\left(x\right)=-I^{\pm }\left(x\right)\partial_{x}f\left(x\right)/f\left(x\right)$ . Comparison with Eq. (A.3) results in [60]

(A.4)
$$a_{\text{t}}^{\pm }\left(x\right)=\pm \frac{\partial_{x}f\left(x\right)}{f\left(x\right)}.$$

Thus, the taper is represented in the 1D model as an effective loss coefficient in one direction and an effective gain coefficient in the other direction. For an adiabatic linear taper, which widens respectively narrows the waveguide by a factor $\alpha =f\left(x_{2}\right)$ , as used in the main text of the paper, we obtain $f\left(x\right)=1+\left(\alpha -1\right)\left(x-x_{1}\right)/\left(x_{2}-x_{1}\right)$ , and thus

(A.5)
$$a_{\text{t}}^{\pm }\left(x\right)=\pm \frac{\left(\alpha -1\right)}{\left(x_{2}-x_{1}\right)+\left(\alpha -1\right)\left(x-x_{1}\right)}.$$
